# Electroformation of Giant Vesicles on a Polymer Mesh

**DOI:** 10.3390/membranes1030184

**Published:** 2011-07-15

**Authors:** Yukihisa Okumura, Takuya Sugiyama

**Affiliations:** Department of Chemistry and Material Engineering, Faculty of Engineering, Shinshu University, Wakasato, Nagano 380-8553, Japan; E-Mail: tsugiya@mc84.shinshu-u.ac.jp

**Keywords:** electroformation, electroswelling, giant vesicles, giant liposomes, lipid membrane, polymer mesh, immobilized vesicles

## Abstract

Electroformation of cell-sized lipid membrane vesicles (giant vesicles, GVs) from egg yolk phosphatidylcholine under applied electric voltage was examined on a substrate of a polymer mesh placed between two planar indium tin oxide coated glass electrodes. Under appropriate conditions, GVs were formed in good yield on meshes of various polymer materials, namely, hydrophobic poly(propylene), poly(ethylene terephthalate), a carbon fiber/nylon composite, and relatively hydrophilic nylon. Arranging threads in a mesh structure with appropriate openings improved GV formation compared to simply increasing the number of threads. For optimal electroformation of GVs, the size and shape of a mesh opening were crucial. With a too large opening, GV formation deteriorated. When the sides of an opening were partially missing, GV formation did not occur efficiently. With an adequate opening, a deposited lipid solution could fill the opening, and a relatively uniform lipid deposit formed on the surface of threads after evaporation of the solvent. This could supply a sufficient amount of lipids to the opening and also prevent a lipid deposit from becoming too thick for electroformation. As a result, good GV formation was often observed in openings filled with swelled lipid.

## 1. Introduction

Giant lipid membrane vesicles (GVs) have been used as model membranes in various biophysical/biochemical studies or in the construction of membrane-based microchemical systems [1,2]. Among the various preparation methods of GVs [2], a procedure that uses applied electric voltage to regulate lipid swelling is known as electroformation or electroswelling and has frequently been used [2,3]. The protocol is relatively simple and may form many well-shaped GVs (typically 10–100 μm in diameter) at a time.

In application of liposomes, immobilization is an important technique. Immobilization of liposomes on an appropriate substrate greatly improves their handling. For example, in place of gel filtration or ultracentrifugation, which needs significant time and costs, immobilized liposomes may be separated from an aqueous bulk phase by ordinary filtration or sedimentation with low-speed centrifugation. Previously, small liposomes (diameter <1 μm) were immobilized on cross-linked polymer gel particles that were chemically modified to have hydrophobic moieties [4,5,6]. Using the immobilized liposomes, Lundahl and coworkers developed liposome chromatography [4]. Also, Khaleque and his coworkers reported gel particles that could reversibly immobilize small liposomes [5,6].

An advantage of electroformation is that it yields immobilized GVs. The formed GVs are ready for microinjection [7]. Also, the bulk aqueous phase may be conveniently replaced by using a flow cell [8,9,10,11]. For example, Estes and his coworker used a flow chamber to obtain GVs in a solution of high ionic strength to investigate binding of a protein to membrane under physiological conditions [8]. Also, in the construction of oligovesicular vesicles with heterogeneous inner membrane-separated microcompartments, which could be an advanced model membrane of biological cells, the composition of aqueous phases was controlled by the replacement in a flow cell [11].

In ordinary electroformation, GVs are formed from a thin lipid layer deposited on an electrode and may therefore be immobilized only on an electroconductive material. Previously, the authors demonstrated that GVs could be produced on a substrate placed between the two electrodes [12]. The substrate may be a non-electroconductive material such as a borosilicate glass tube. This greatly increases the number of materials usable for a substrate that holds electroformed GVs.

At the same time, the study also revealed a limitation of the procedure. Electroformation of GVs does not occur efficiently on a planar material without openings such as a solid glass plate. Lipid deposited on the central part of such a substrate did not respond to the applied electric field, and GVs were formed only near or at the edge of the substrate [12]. In this respect, to produce many immobilized GVs on a substrate, a mesh could be useful. Particularly, meshes made of polymer materials are of interest because they are widely available and inexpensive. They are also chemically inert in a standard neutral aqueous environment. The previous study only briefly showed a preliminary result of electroformation on a poly(ethylene terephthalate) (PET) mesh. In the present study, we investigated electroformation on meshes of various polymer materials, PET, nylon, poly(propylene) (PP), and a carbon fiber/nylon composite, with different mesh openings.

## 2. Experimental Section

### 2.1. Materials

Phosphatidylcholine extracted and purified from egg yolk (eggPC) was purchased from Avanti Polar Lipids (Alabaster, AL, USA). The phospholipid was checked by using thin layer chromatography on a silica gel plate (Silicagel 70 Plate-Wako from Wako Pure Chemicals (Osaka, Japan)) developed in chloroform/methanol/water (65:25:4 v/v/v), and only a single spot was seen. Indium tin oxide coated glass (ITO-glass) was obtained from AGC Techno Glass Co., Ltd. (Funabashi, Chiba, Japan). Methanol was of the analytical grade and a product of Wako Pure Chemicals. PET (PETEX^®^), nylon (NYTAL^®^), poly(propylene) (PROPYLTEX^®^), and carbon fiber/nylon composite (CARBOTEX^®^) meshes were products of SEFAR AG (Heiden, Switzerland).

### 2.2. Electroswelling of Lipid on a Mesh

**Figure 1:**
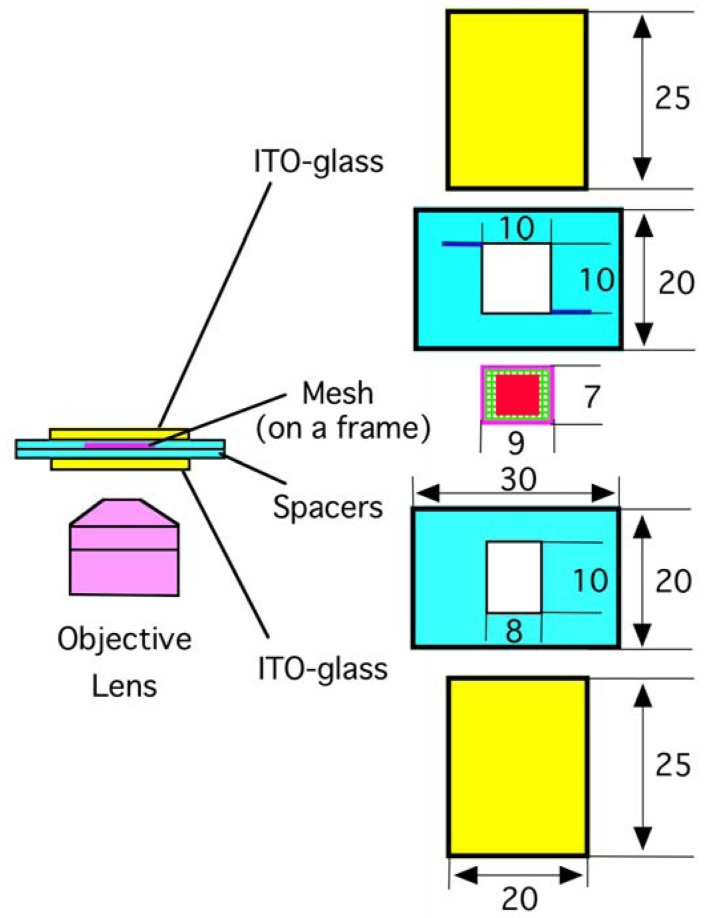
An electroformation chamber with a mesh substrate. A small piece of a polymer mesh with a deposit of egg yolk phosphatidylcholine (shown as a red square) was placed at the median between the two electrodes. Two thin channels were carved on the upper spacer (shown as two blue lines) for the introduction of ultrapure water into the chamber. The unit of length is millimeter. ITO: Indium tin oxide coated glass.

An electroformation chamber was assembled using adhesive tapes as schematically shown in Figure 1. Two planar ITO-glass electrodes (20 mm × 25 mm, thickness 1 mm) and two polystyrene spacers (20 mm × 30 mm, thickness 0.5 mm) were stacked. The electroconductive surfaces of the ITO electrodes were placed facing each other. A small piece of a polymer mesh (7 mm × 9 mm) was attached onto a supporting frame made of a thin piece of PET sheet (outer size 7 mm × 9 mm, inner opening 6 × 8 mm, 0.2 mm thickness). The frame was placed on the lower spacer, and the chamber was thoroughly washed with Milli-Q grade ultrapure water and dried. A methanolic solution of eggPC (5.0 or 10.0 mg/mL, 1.0 μL) was deposited in the area of 6 mm × 6 mm on the mesh. Meshes seemed to tolerate the very brief exposure to methanol. There was no detectable change in the appearance of the mesh fibers before and after the exposure. The chamber was further dried under reduced pressure produced with a water aspirator. Ultrapure water was gently introduced into the chamber through one of the two thin channels carved on the upper spacer, and sinusoidal ac voltage (5.0 Vpp (peak-to-peak), 2 Hz) was applied between the electrodes from a function generator (Kenwood TMI FG-272, Yokohama, Japan). Lipid swelling was monitored on an inverted optical microscope equipped with phase contrast and digital image enhancement options (Olympus IX-50, Tokyo, Japan).

For the calculation of a GV formation index, openings with the deposited lipid were randomly taken (typically, 20–30 openings). For each opening, the percentage of the lipid area seen with spherical GVs larger than 10 μm at the part of the lipid close to the objective lens was determined, and the values were averaged for all the openings examined.

In experiments with polymer threads, threads were carefully removed from a mesh, and a pair of the threads or a partially broken mesh was attached onto the supporting frame. A lipid solution was then deposited on the threads (5.0 mg/mL, 0.5 μL) or on the broken mesh (5.0 mg/mL, 1.0 μL).

## 3. Results and Discussion

### 3.1. Electroformation on a PET Mesh

**Figure 2:**
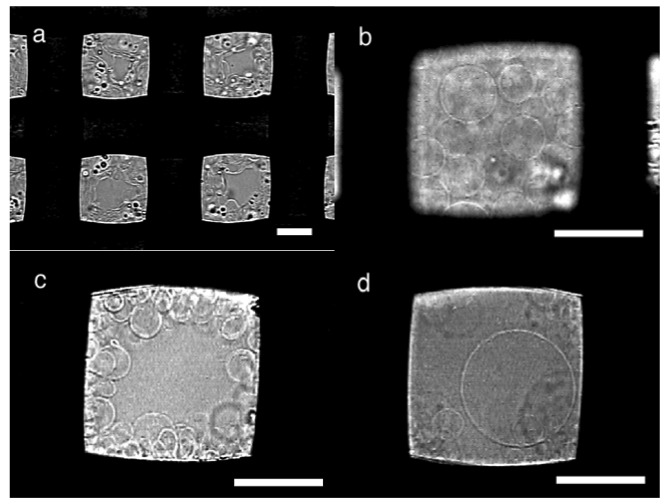
**(a)** Electroswelling of lipids on a PET mesh #145. The image was taken 3 min after application of sinusoidal ac voltage (5.0 Vpp, 2 Hz); **(b)** GVs seen in an opening filled with swelled lipids at 90 min; **(c)** GVs formed on the threads at 80 min; **(d)** A large GV observed at 100 min. Bar = 50 μm.

In typical electroswelling on a PET mesh (#145, nominal opening 105 μm, thread diameter 77 μm) as a substrate, upon application of ac voltage, a deposit of egg phosphatidylcholine (eggPC; 10 μg) on the mesh immediately started swelling (Figure 2a). During the swelling, the lipid vibrated in synchronization with the oscillation of the applied electric voltage as previously observed in electroformation on a platinum wire electrode or substrate [7,12,13]. In the early stage, the swelled lipid layer formed semi-spherical domes, and the domes gradually grew to GVs. With plenty of lipids, the swelled lipid covered openings of the mesh in approximately 20 min, and later, spherical GVs were seen in the openings (Figure 2b). The GVs were stacked and formed a layer of approximately 80 μm thickness (estimated from the focal distance of microscopic observation), which was close to the diameter of the threads. In openings with less lipids, GVs were seen on the sides (Figure 2c). The electroformation was usually completed in 80–100 min. GV formation was evaluated by determining the approximate percentage of the swelled lipids covered with GVs in the openings (GV formation index). With a PET mesh #145, the typical index value was 80–90% at the end of electroformation.

The openings covered with swelled lipids usually yielded relatively large GVs. In typical formation, many GVs had the diameter of 15–40 μm (Figure 2b). Some GVs were as large as 60 μm (Figure 2d) although, in the present case, no GV larger than the opening (105 μm) was observed. In contrast, in openings with less lipids, only GVs smaller than 20 μm were usually seen (Figure 2c).

When a PET mesh with a lipid deposit was left in pure water without electric voltage, spontaneous swelling occurred. However, in this case, only a small number of GVs were found along with other non-spherical membranous structures such as myelins or mushroom-like objects.

PET meshes of five different mesh numbers that had various opening sizes and thread diameters were tested for a substrate of electroformation, and the results were summarized in Table 1. Among the meshes examined, the optimal GV formation was seen when a mesh #145 was used with 10 μg of a lipid deposit. Two meshes of the higher mesh numbers (#287 and #198) also yielded GVs efficiently. On the two meshes, most of the openings were covered with swelled lipids, and GV formation occurred in a manner similar to the mesh #145.

**Table 1:** GV formation on PET meshes with sinusoidal ac voltage (5.0 V, 2 Hz).

**Mesh number**	**Opening size [μm]**	**Thread diameter [μm]**	**Lipid deposit [μg]**	**GV formation index [% ]**	**Typical diameter of GVs [μm]**
#287	51	38	10	60–70	10–40
#198	74	54	10	70–80	10–40
			5	80	10–40
#145	105	77	10	80–90	15–40
			5	30–40	15–50
#102	150	96	10	10	20–40
#71	210	147	10	< 10	n/a

Meshes of the lower mesh numbers (#102 and #71) showed poor GV formation. In those meshes, no opening was filled with swelled lipids. GVs were formed on approximately only 10% of the lipid deposit seen in the openings. Instead, many distorted vesicles and membranous objects of irregular shapes were observed.

With a mesh #145, the GV formation index decreased significantly when the amount of deposited lipids was reduced from 10 μg to 5 μg. In the latter case, the electroformation resembled that on a mesh with the lower mesh number. Most of the openings were not filled with swelled lipids. These results indicate that the mesh number and the distribution of deposited lipids on mesh threads should be crucial for efficient GV formation.

A crude estimation shows that the surface area of a mesh usually increases as the mesh number becomes lower (calculations shown in Supplementary Materials). With a same amount of lipids deposited, a mesh of the lower number should therefore have a smaller amount of lipids per surface area. Since GV formation deteriorates with less lipids as observed in the case of a mesh #145, this could be a reason for the inferior GV formation at the lower mesh numbers. However, the estimated surface area of a mesh #102 is only 15% larger than that of a mesh #145, and the difference is probably too small to solely explain the large gap in the GV formation index observed between the two meshes (10% for #102 and 90% for # 145 with 10 μg of deposited lipids).

### 3.2. Electroformation on a Nylon Mesh

**Figure 3:**
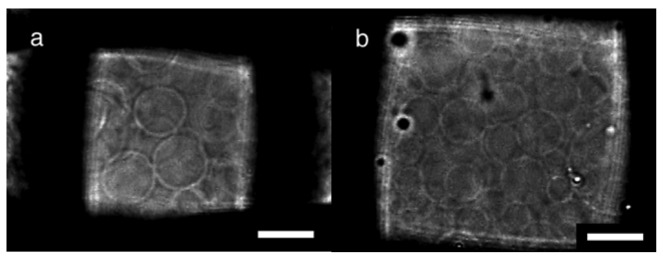
**(a)** GV formation on a nylon mesh #196 with sinusoidal ac voltage (5.0 Vpp, 2 Hz) at 70 min; **(b)** A nylon mesh #145 at 100 min. Bar = 20 μm.

Nylon meshes were also tested for a substrate of electroformation, and the typical results under the optimal conditions are summarized in Table 2. The process of the GV formation was essentially the same as with PET meshes. With 10 μg of deposited lipids, the GV formation indexes were high in the cases of nylon meshes #307 and #196, which had the opening size of approximately 60 μm (Figure 3a). PET meshes of the comparable openings also showed good GV formation (Table 1). Meanwhile, on nylon meshes #170 and #145, the optimal GV formation occurred when those meshes were used with a smaller amount of lipids (5 μg). A nylon mesh with large openings (#70) was as a poor substrate as a PET mesh of a similar mesh number (#71).

**Table 2:** GV formation on nylon meshes with sinusoidal ac voltage (5.0 V, 2 Hz).

**Mesh number**	**Opening size [μm]**	**Thread diameter [μm]**	**Lipid deposit**	**GV formation index [% ]**	**Typical diameter of GVs [μm]**
#307	53	33	10	70–80	10–40
#196	65	65	10	60–70	15–60
#170	75	77	5	80–90	10–50
#145	105	77	5	80–90	10–50
#70	210	155	10	< 10	10–30

Assuming uniform distribution of lipids all over the surface of mesh threads, a mesh #70 should have almost the same estimated amount of lipids per surface area as a mesh #145 (see Supplementary Materials). However, the two meshes were largely different in the observed GV formation index. The result suggests possible non-uniform distribution of the lipids and/or a significant effect of the geometry of the threads in a mesh on GV formation.

Although the electroswelling process was essentially the same between nylon and PET, there also was a small but noticeable difference in GV formation. The optimal formation for a nylon mesh #145 occurred with less lipids than PET #145 although both meshes had the same opening size and thread diameter.

### 3.3. Electroformation and the Geometry of Threads

**Figure 4:**
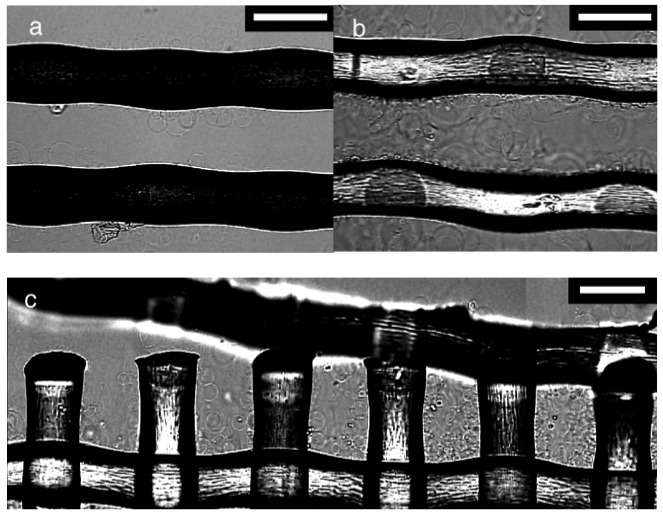
**(a)** Electroswelling on a pair of two parallel threads taken from a PET mesh #145 with sinusoidal ac voltage (5.0 Vpp, 2 Hz) at 90 min; **(b)** A pair of two parallel threads from a nylon mesh #145 at 100 min; **(c)** Electroswelling at a partially broken edge of a nylon mesh #145 at 100 min. The picture is a composite of three frames separately taken. Bar = 100 μm.

To look further into the effect of the mesh number and the observed difference between PET and nylon, electroformation was examined on polymer threads. A pair of threads was isolated from a mesh of PET #145 or nylon #145, and used as the substrate. The GV formation on those threads (Figure 4) was inferior to that observed on their parent meshes. In the case of PET, not many swelled lipids or GVs were observed although the threads should have possessed a sufficient amount of lipids on their surfaces. The observation suggests that a large part of the lipid deposit should have been on a non-observable part of the threads and formed a thick layer. When PET (Figure 4a) and nylon (Figure 4b) were compared, the latter had more visible lipids and GVs, indicating relatively uniform distribution of the lipids over the thread surface. The swelled lipids almost filled the gap between the two parallel nylon threads, and the swelling resembled that usually occurring in an opening of the parent mesh.

In Figure 4c, electroswelling of lipids at a partially broken edge of a nylon mesh #145 was shown. The swelled lipids filled an intact opening seen in the right part of the picture. A smaller amount of lipids were visible as the openings became less complete toward the left. The result indicates that the geometry of threads affects the distribution of lipids and GV formation on a mesh.

These observations suggest a possible explanation for the effect of the mesh number on GV formation. As shown by the above experiment with threads, the distribution of deposited lipids on the thread surface is not uniform. When a lipid solution is applied to a mesh, the solution fills the openings. Evaporation of the solvent leaves a sufficient amount of lipids in the opening to fill it upon swelling. As a result, good GV formation is often observed in those openings filled with swollen lipids. The filling occurs more frequently with smaller openings. If an opening is too large the lipid solution cannot be held in the opening, and most of the solution stays on the bottom surface of the threads due to gravity. This results in the formation of a thick lipid layer on the non-observable part of the thread. Such a thick layer is unsuitable for electroformation. Previously, Angelova showed that GV formation significantly deteriorated on a Pt wire electrode when a lipid layer was too thick [14]. Considering the results with PET and nylon meshes, the filling often seems to occur when the mesh opening is smaller than approximately 100 μm.

The geometry of threads could also affect GV formation through filling with a lipid solution. An incomplete opening could not hold the deposited solution well. The result with a partially broken mesh is consistent with this view. Arranging threads in a mesh structure with an appropriate size of openings may yield more GVs compared to simply increasing the number of threads.

As far as the filling occurs, a mesh with larger openings may be preferable for GV formation. A planar substrate with no openings does not efficiently produce GVs [12]. Therefore, there should be a certain minimum measurement of the opening size that allows GV formation, even though this point was not clearly seen with the meshes tested in the present study. Also, in a small opening, GV formation could be limited by crowding. Sometimes, GVs larger than an opening of a mesh were formed but only on the outside surface of the mesh. In addition, considering the estimated surface area (see Supplementary Materials), lipids that could be loaded on a mesh without making the deposit too thick for electroformation should be less for a mesh with smaller openings. With the same GV formation index, the actual number of formed GVs is larger when more lipids are present.

Another factor that may affect the lipid deposition is the wetting characteristics of a polymer thread with a lipid solution. The difference between PET and nylon in the optimal amount of a lipid deposit may be attributed to the relatively more uniform spreading of the solution on the former, possibly due to the higher hydrophilicity (water contact angles, 63.1 degrees for nylon and 70.2 for PET) [15].

### 3.4. Electroformation on Meshes of Poly(propylene) and a Carbon Fiber/Nylon Composite

**Figure 5:**
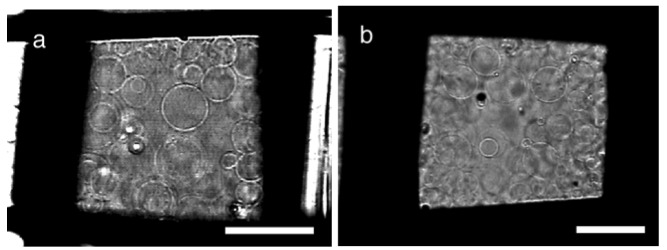
**(a)** Electroformation of GVs on a poly(propylene) mesh #121 with sinusoidal ac voltage (5.0 Vpp, 2 Hz) at 60 min; **(b)** GVs formed on a mesh of a carbon fiber/nylon composite #145 at 80 min. Bar = 50 μm.

Electroformation was also tested with meshes of two other polymer materials, poly(propylene) (PP) and a carbon fiber/nylon composite, as a substrate. On both polymer meshes, GV formation occurred in a manner similar to that on PET or nylon in good yields (Figure 5). Typical results under optimal conditions are shown in Table 3.

The results showed that the electroformation on a substrate could be used with various polymers, from hydrophobic poly(propylene) to relatively hydrophilic nylon (water contact angle of PP, 96.9 degrees) [15]. Naturally, the optimal mesh number could differ depending on the actual material used but the opening size of approximately 100 μm seems to yield good GVs.

**Table 3:** GV formation on other polymer meshes with sinusoidal ac voltage (5.0 V, 2 Hz).

**Materials**	**Mesh number**	**Opening size [μm]**	**Thread diameter [μm]**	**Lipid deposit [μg]**	**GV formation index[%]**	**Typical diameter of GVs [μm]**
Poly(propylene)	#121	105	106	10	80	20–50
Carbon fiber/nylon	#145	130	52	10	90	15–50

## 4. Conclusions

The present study demonstrated that electroformation of GVs could occur on meshes of various polymer materials. The geometry of mesh threads, especially the size of the openings significantly affected the GV formation. For electroformation of many GVs on a polymer substrate, arranging threads in a mesh structure is preferable to simply using multiple unorganized threads. By choosing a mesh of appropriate openings, GVs may efficiently be obtained.

The procedure yields electroformed GVs immobilized on a desired polymer material. If an application is cost-sensitive, one could use an inexpensive polymer mesh such as the one used in the present study. The electrodes, which often incur a large part of the costs, could then be reused. Or, by using a functionalized fiber as a substrate, one could conveniently produce a composite of the fiber with GVs.

Another advantage of the procedure is that it may be effective in avoiding an inadvertent electrochemical process at electrodes. In reconstitution of redox-sensitive functional molecules into GVs through electroformation, the control of electrochemical reactions should be important. Previously, peroxidation of unsaturated lipids on an ITO-coated electrode during electroformation was reported [16]. A recent review by Walde and his coworkers suggested that the present procedure could be a possible solution to the problem [2]. Although electroswelling on a negative electrode with dc voltage may also prevent the lipid peroxidation [17], the present procedure separates the place of GV formation from electrodes and may be useful to avoid other electrochemical reactions.

## Supplementary Files

Supplementary File 1 Supplementary Materials (PDF, 107 KB)
