# Supplementary material for: Electroformation of Giant Vesicles on a Polymer Mesh
Source: Membranes (Basel). 2011 Jul 15;1(3):184–94. doi: 10.3390/membranes1030184 (PMC4021901; doi:10.3390/membranes1030184)
Supplement: Supplementary File 1 — Supplementary Materials (PDF, 107 KB) [file membranes-01-00184-s001.pdf]

Supplementary Materials

An Estimation of the Surface Area of a Mesh

By ignoring the crossing points of threads and representing threads with cylinders, the number of hypothetical cylindrical threads in a square mesh (N) should approximately be 2 × L/(A + D), where L is the size of the mesh, A, the size of an opening, and D, the diameter of a thread. The surface area of a cylindrical thread (S) is $4 \times 3.14 \times (D/2)^2$, ignoring the cross section of the thread. The total surface area of the square mesh is given by N × S.

Based on the model, the surface area of the PET and nylon meshes used in the present study can be calculated and shown in Tables S1 and S2, respectively. Uniform distribution of the lipid all over the surface of threads was assumed. Although PET #145 and #102 should be similar in the estimated surface area and the amount of lipid per area, they are different in the GV formation index. Similarly, nylon #145 and #70 nominally had the almost same amount of lipid per area, but #70 showed poor GV formation. These results suggest that the actual amount of lipid per area should be largely different from the estimation and/or that the geometry of threads in a mesh should have a significant effect on GV formation.

Table S1. The surface area of PET meshes (with 10 μg lipid deposited).

Mesh number	Opening size [μm]	Thread diameter [μm]	Surface area [mm^2]	Lipid deposit per area [$μg/mm^2$]	GV formation index [%]
#287	51	38	0.61	16	60–70
#198	74	54	0.86	12	70–80
#145	105	77	1.23	8.1	80–90
#102	150	96	1.41	7.1	10
#71	210	147	2.28	4.4	<10

Table S2. The surface area of nylon meshes.

Mesh number	Opening size [μm]	Thread diameter [μm]	Surface area [mm^2]	Lipid deposit [μg]	Lipid deposit per area [$μg/mm^2$]	GV formation index [%]
#307	53	33	0.48	10	21	70–80
#196	65	65	1.22	10	8.1	60–70
#170	75	77	1.47	5	3.4	80–90
#145	105	77	1.23	5	4.1	80–90
#70	210	155	2.48	10	4.0	<10

© 2011 by the authors; licensee MDPI, Basel, Switzerland. This article is an open access article distributed under the terms and conditions of the Creative Commons Attribution license (http://creativecommons.org/licenses/by/3.0/).
